# Oral uricase eliminates blood uric acid in the hyperuricemic pig model

**DOI:** 10.1371/journal.pone.0179195

**Published:** 2017-06-08

**Authors:** Paulina Szczurek, Nadia Mosiichuk, Jarosław Woliński, Tetiana Yatsenko, Danica Grujic, Liudmyla Lozinska, Marek Pieszka, Ewa Święch, Stefan Grzegorz Pierzynowski, Kateryna Goncharova

**Affiliations:** 1 Department of Biology, Lund University, Lund, Sweden; 2 Department of Animal Nutrition and Feed Sciences, National Research Institute of Animal Production, Balice, Poland; 3 Department of Biochemistry and Biotechnology, Vasyl Stefanyk Precarpathian National University, Ivano-Frankivsk, Ukraine; 4 Department of Endocrinology, The Kielanowski Institute of Animal Physiology and Nutrition, Polish Academy of Sciences, Jabłonna, Poland; 5 Allena Pharmaceuticals, Newton, Massachusetts, United States of America; 6 Department of Monogastric Nutrition, The Kielanowski Institute of Animal Physiology and Nutrition, Polish Academy of Sciences, Jabłonna, Poland; 7 Anara AB, Malmo, Sweden; 8 Institute of Rural Health, Lublin, Poland; University Medical Center Utrecht, NETHERLANDS

## Abstract

An elevated level of serum uric acid—hyperuricemia, is strongly associated with the development of gout and chronic kidney disease (CKD) which is often accompanied by a significantly reduced glomerular filtration rate (GFR). In the present study, we investigated the extra-renal elimination of uric acid via the intestine in a healthy pig model and the effect of oral uricase therapy on plasma uric acid concentrations in pigs with induced hyperuricemia and CKD. The experiment was conducted on eleven, ten-week-old pigs (n = 11). The porcine model of CKD was developed by performing 9/10 nephrectomy surgery on eight pigs. A stable model of hyperuricemia was established in only five of the eight nephrectomized pigs by frequent injections of uric acid (UA) into the jugular vein. All pigs (three healthy pigs and five CKD pigs) were operated for implantation of jugular vein catheters and the three healthy pigs also had portal vein catheters inserted. Blood uric acid concentrations were measured spectrophotometrically, using the Uric Acid Assay Kit (BioAssay Systems, Hayward, USA). The piglets with CKD received orally administered uricase (treatment) and served as their own controls (without uricase supplementation). Oral uricase therapy significantly decreased plasma uric acid concentrations in pigs with CKD, whereas hyperuricemia was observed in the pigs whilst not being treated with uricase. Urinary uric acid excretion was similar during both the treatment and control periods during the first 8 h and 24 h after UA infusions in the CKD pigs. To demonstrate the elimination of UA via the intestine, the healthy pigs were infused with UA into the jugular vein. The blood collected from the jugular vein represents circulating UA concentrations and the blood collected from the portal vein represents the concentration of UA leaving the intestine. The final (after 2 h) concentration of UA was significantly lower in blood collected from the portal vein compared to that collected from the jugular vein (3.34 *vs*. 2.43 mg/dL, respectively, *p* = 0.024). The latter allows us to suggest that UA is eliminated from the blood via the gut tissue.

## Introduction

Uric acid (UA) is a poorly soluble final product of purine metabolism in humans. Most mammals express urate oxidase (uricase) which converts UA to the more soluble allantoin [1]. However, due to nonsense mutations in the uricase gene in humans, great apes and several other species, ‘uricase knockouts’, are unable to degrade UA. Under physiological conditions the majority of UA circulates in the plasma in a free form, as ionized urate salt. About two-thirds of the daily UA pool is primarily excreted by the kidneys, but more than 90% of this amount is reabsorbed by transporters in the proximal renal tubule [2]. Only 10% of the daily UA pool is then excreted from the kidneys via the urine. The remaining one-third is cleared through the gastrointestinal tract (GIT), where urate can be eliminated by gut bacteria in a process called uricolysis [3]. Plasma UA concentration depends on the balance between UA generation and excretion, as well as purine *de novo* synthesis, catabolism and turnover [4]. Both increased production of UA and/or its impaired excretion may lead to hyperuricemia (HUA), a condition defined by a plasma UA concentration above 6.8 mg/dL [5]. Due to the lack of uricase and the reabsorption of UA in the kidney proximal tubule, UA concentrations in humans are much higher in comparison to that of other mammals and moreover, they can be easily modified by diet. The consumption of alcohol, dietary purines (meat, seafood, offal) and products with high fructose content are possible risk factors for the development of HUA and gout. There is evidence to suggest that some genetic factors may also contribute to the development of HUA, specifically those involving polymorphisms in urate transporters [6].

Three main pharmacological treatment strategies are currently used in the treatment of HUA. The first treatment strategy is aimed at lowering UA generation and includes the use of xanthine oxidase inhibitors such as allopurinol and febuxostat. However, their effectiveness in lowering serum urate concentrations is limited, since only between 30–60% of patients receiving these therapies reach a plasma UA concentration of < 6 mg/dL [7]. Moreover, allopurinol is eliminated by the kidneys, and thus patients with chronic kidney disease (CKD) and thus impaired GFR may be at an increased risk of its toxicity [2]. The second treatment strategy is based on the use of uricosuric agents, such as probenecid and benzbromarone, which promote the excretion of UA in the urine. Unfortunately, these drugs can increase the crystallization of UA, inducing the formation of kidney stones and/or the development of hepatotoxic effects. The last treatment strategy includes the use of exogenous uricases, which metabolize UA to allantoin. Allantoin is 5–10 times more soluble than UA, and thus is more readily eliminated [4]. Clinical trials have shown that microbial uricase is more effective for the treatment of HUA and gout than allopurinol [8]. A recombinant form of uricase, rasburicase, is however characterized by a short half-life and high immunogenicity. Other uricase preparations, such as pegloticase, may be easily deactivated and physically unstable [3]. Thus, due to the undesirable biological properties and possible side effects of microbial uricase intravenous infusions, their use is very limited.

Thus, the majority of the currently approved therapies for HUA and gout are not recommended for patients with late-stage chronic renal disease. There is therefore a need for alternative, safe and well tolerated therapies which are effective in reducing plasma UA concentrations in patients with late stages of CKD.

Based on the peculiarities of urate metabolism and the fact that one third of the daily urate pool is excreted via the small intestine, we were interested in developing an oral enzyme therapy using uricase for patients that have HUA and late stages of CKD. For this purpose we chose to make use of the pig model, as it is widely used in translational research, surgical models, and procedural training in preclinical toxicological testing of pharmaceuticals [9]. However, pigs, as well as other non-primates, demonstrate low endogenous plasma urate concentrations (1.2–1.6 mg/dL) due to the presence of uricase, which converts uric acid to allantoin in the liver. Therefore, to increase plasma UA concentrations in the pig we infused them with a UA suspension.

In the present study, we first aimed to develop a reliable pig model for investigation of the extra-renal elimination of UA through the intestine. Secondly, we wanted to investigate the ability of orally administered uricase, from *Candida utilis*, in decreasing plasma UA concentrations in CKD pigs with induced HUA. We hypothesized that oral uricase therapy may be effective in decreasing serum UA concentrations in CKD pigs, as a result of enhanced intestinal urate elimination.

## Materials and methods

This study was carried out in strict accordance with the recommendations in the Guide for the Care and Use of Laboratory Animals of the National Institutes of Health. The original protocol for this study is available as supporting information; see S1 Study Protocol. All experimental procedures were approved by the Malmö –Lund, Local Ethical Review Committee for Animal Experiments (Permit Number: M73-15). All efforts were made to minimize animals’ suffering.

### Animals and diets

The experiment was performed on 11 castrated male pigs ((Swedish Landrace X Yorkshire) x Hampshire) with an average initial body weight of 12 ± 3 kg and an average age of 10 ± 2 weeks at the beginning of the experiment. The pigs were selected from a specific pathogen-free herd (Vindfälle 810, 268 68 Röstånga, Sweden). The pigs were divided into two groups–the healthy group (n = 3) and the CKD group (n = 8). Pigs were housed in individual metabolic cages equipped with a dry feeding trough, a drinking nipple and a constant heating lamp (150 W). The metabolic cages allowed for the collection of urine. Pigs were allowed to move freely within their cages and had visual contact with each other. Pigs were maintained on a 12 hour day-night cycle at a constant temperature (22 ± 2°C). Pigs were fed twice daily (2% of their b.w.) with cereal-based feed (“Morawski”, Żurawia, Poland), with a low calcium (0.09%) and high fructose (20%) concentration. The feed was enriched with inosine (4% of total food amount/day) to induce HUA. This amount of feed is comparable to the amount ingested when pigs are given *ad libitum* access to feed under similar conditions. During enzyme treatment, the feed was also supplemented with sodium bicarbonate (1% of total food amount/day). Water supply was limited and corresponded to an amount of between 10–12% of the pigs’ body weight. During the experimental period food and water intake was measured daily. Pig body weight was measured weekly.

### Surgery

Prior to surgery pigs were fasted overnight and pre-medicated with azaperone (Stresnil, Janssen Pharmaceutica, Beerse, Belgium, 4.0 mg/kg i.m.). The pigs were then anaesthetized with 2-bromo-2-1.1.1-triflouroethane (Fluothan, Astra Läkemedel, Södertälje, Sweden), mixed with air and O_2_ as a carrier gas, at approximately 0.5–1 L/min in a close-circuit respiratory system (Komesaroff Medical Developments, Melbourne, Australia).

To develop CKD in the pigs, 8 pigs underwent 9/10 nephrectomy surgery. The nephrectomy was achieved by renal tissue infarction. The main left renal artery was completely ligated using sterile silk sutures which resulted in the blockage of blood flow to the entire left kidney. The right renal arterial branches were ligated successively in order to achieve an obstruction to blood flow to 90% of the right kidney. As a result, discoloration of the entire left kidney and 90% of the right kidney was observed, confirming the infarction.

In all pigs (healthy and CKD pigs; n = 11) the left external jugular vein was catheterized using silicon tubing (Helix Medical Carpinteria, CA, USA) with an outer diameter of 1.64 mm and an inner diameter of 0.75 mm. The catheter was exteriorized percutaneously on the dorsal side of the neck.

For investigation of the extra-renal elimination of UA through the intestine, the three healthy pigs had an additional catheter inserted into the portal vein, just prior to the entrance to the liver. The portal vein was catheterized using silicon tubing (Silastic Laboratory Tubing, Dow Corning, Auburn MI, USA) with an outer diameter of 2.29 mm and an inner diameter of 1.27 mm. Briefly, after localization of the portal vein in the liver hyllus, a double portabac silk (Ethicon SILK 5–0, Lidingö, Sweden) suture (diameter 0.5 cm) was placed on the portal vein wall. A small incision was made in the middle of the portabac suture, then a catheter with fixative cuffs was inserted into the portal vein for 3–4 cm up to the first cuff towards the liver. The portabac suture was then closed over the catheter and the bleeding stopped. Finally, the cuff was gently sutured (Ethicon SILK 5–0, Lidingö, Sweden) to the portal vein wall. Another 3–4 cuffs were placed on the catheter and the catheter was then fixed onto the inside of the abdominal cavity. The catheter was exteriorized using a surgical needle on the right flank of the abdominal wall. A single external cuff protected the catheter from being drawn back into the abdominal cavity. After the surgical procedures, for 3 days following surgery, the pigs received 1.5 mL of Buprenorphine (“Vetergesic”, Alstoe Ltd., York, UK).

The pigs were allowed a 2-week recovery period before the experimental period commenced.

### Experimental design

The current study was comprised of two experiments. In the first experiment the intestinal elimination of UA was investigated in the three healthy pigs (average body weight of 15 ± 3 kg), on two separate occasions with a 1-week interval between the two assessments. Each pig underwent surgery to have a catheter inserted into the jugular vein and one into the portal vein. The blood collected from the jugular vein represents the dynamics of the peripheral circulation, whereas the blood collected from the portal vein represents the dynamics of the intestinal circulation. To reach saturation threshold for UA, the pigs were intravenously infused, via the jugular vein catheter, with a UA suspension (40 mg/mL in 40% glucose, pH 7.0, at dose of 5 mg/kg b.w.) every 30 min during a two hour period; the pigs received four infusions in total. Blood samples were collected from both the jugular and portal veins at baseline (before UA infusions) and thereafter repeatedly at 1 and 2 h following the UA infusion. Urine was collected twice in the 24 h period.

The second experiment was performed on five nephrectomized pigs (which were selected from the eight pigs that underwent 9/10 nephrectomy surgery based on elevated plasma creatinine concentrations indicating successful development of CKD), with an average body weight of 16 ± 3 kg at the beginning of the experiment. To induce HUA, pigs were i.v. infused with a UA suspension (40 mg/mL in 40% glucose, pH 7.0 at dose 5 mg/kg b.w.) every 30 min for an eight hour period–with 16 infusions in total. Uricase from *Candida utilis* (25% pure in sodium borate, specific activity ~25 units/mg) was administered orally to each pig, together with the feed (18,000 Unit per portion), 1 h prior to and 3 h after beginning the UA infusions. To ensure enzyme stability in the stomach, the pigs received both food and water supplemented with sodium bicarbonate (1%). Uricase from *Candida utilis* was selected as the lead candidate for potential oral enzyme therapy based on results obtained from previous studies by our lab (unpublished data), as a result of its’ wide pH profile (6–8). The experiment was performed as a two-period, two-sequence crossover study, with the following sequences considered: CT (control-treatment) and TC (treatment-control). The sequences, as well as the treatment/control periods within them were separated by a one-day washout period. Thus, the five CKD pigs were assigned to the CT and then to the TC sequence and served as both the treatment (when receiving orally administered uricase) and control groups (without uricase supplementation), such that each pig served as its own control. Blood samples collected from the jugular vein were collected at baseline (before UA infusions), and then at 2, 4, 6, 8, 10, 12, 16 and 24 h following the first UA infusion. Urine was collected every 8 h during the 24 h period. At the end of experiments the pigs were sedated using azaperone (5 mg/kg, Stresnil, Leo), and euthanized by a single dose of intravenously administered sodium pentobarbiturate (100 mg/kg).

### Blood sample collection and analysis

Blood samples were collected into lithium-heparin tubes (BD Vacutainer®, Franklin Lakes NJ, USA, REF 367884), that were gently inverted several times after collection and stored on ice before being centrifuged at 3000×g, for 15 min at 4°C. Plasma was collected and the samples were stored at -20°C until further analysis. Plasma UA and creatinine concentrations were analyzed spectrophotometrically using the Uric Acid Assay Kit and QuantiChrom™ Creatinine Assay Kit, respectively (BioAssay Systems, Hayward, CA, USA), according to the manufacturer’s protocols.

### Urine sample analysis

To prevent precipitation of UA salts, 1–2 ml of 8 M NaOH was added to the urine collection containers. After each collection, aliquots (about 10 mL) of urine samples were transferred into tubes and stored at –20°C until performing the creatinine assay. UA concentration was measured in fresh urine samples. Before analysis, urine samples were diluted 1/10 with dist H_2_O for UA and 1/20 with 7 mM NaOH for creatinine. Urinary concentrations of UA and creatinine were measured spectrophotometrically using the Uric Acid Assay Kit or QuantiChrom™ Creatinine Assay Kit, respectively (BioAssay Systems, Hayward, CA, USA), according to the manufacturer’s protocols.

### Statistical analysis

All analyses were carried out using Prism, version 5 (GraphPad Software, Inc, San Diego, CA, USA). Statistical significance in Experiment 1 was evaluated using a two-tailed Student’s t-test followed by a Student-Newman-Keuls multiple comparisons test. For Experiment 2, the total area under the curve (AUC) was calculated for plasma uric acid concentration, urine uric acid concentration (measured at 8 and 24 hours after uric acid infusion), as well as creatinine and urate clearance. Treatment efficacy was assessed based on the within-subject differences between the control and treatment periods with regards to the various outcomes investigated, using the customary formula for an unpaired t-test [10]. A pre-test to check the assumption of negligible carryover effects was also performed as recommended [10]. Differences were considered significant if *p* < 0.05.

## Results

### Intestinal elimination of UA in pigs

**Fig 1:**
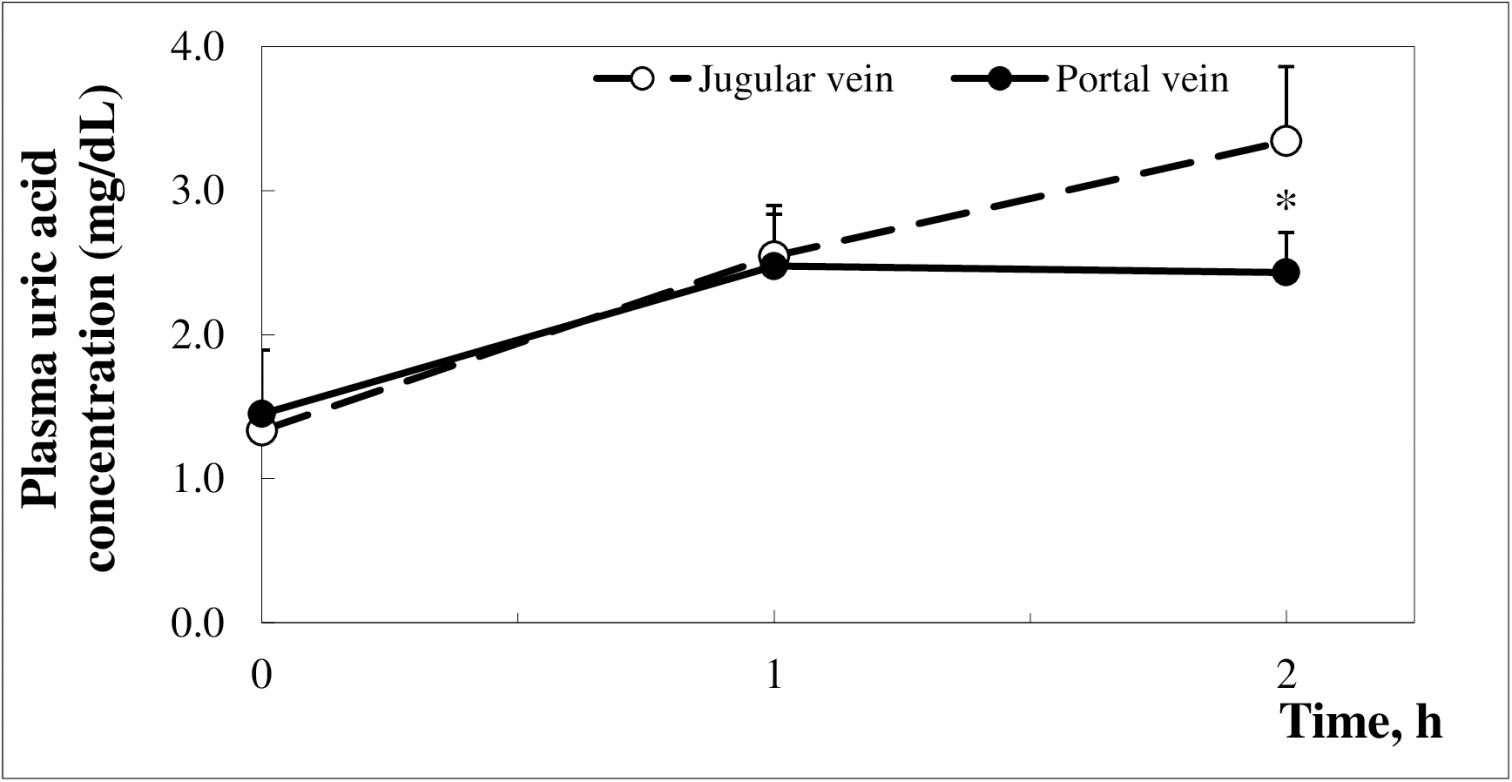
Uric acid concentration in jugular and portal vein blood plasma in healthy pigs i.v. infused with a uric acid suspension (5 mg/kg b.w. each 30 min.), beginning after baseline blood sampling. Data are presented as means ± SD (*n* = 6). Asterix (*) describes a significant difference between uric acid concentrations in portal and jugular vein blood at last point of blood sampling, *p* < 0.05.

Due to the low endogenous plasma urate concentration in pigs (1.2–1.6 mg/dL), in order to reach the saturation threshold for plasma UA the pigs were infused with a UA suspension, via the jugular vein catheter. Fig 1 shows the mean plasma UA concentration in blood collected from the portal and jugular veins of pigs infused a total of four times with the UA suspension (5 mg/kg b.w. each 30 min) in a 2 h period. The basal UA concentration as well as the UA concentration following two i.v. infusions of the UA suspension, were virtually the same in plasma from the portal and jugular vein blood. The first two i.v. infusions of the UA suspension resulted in a 1.6-fold increase in plasma UA concentration in the portal and jugular vein blood compared to basal levels. The following two i.v. UA infusions resulted in a 2.4-fold increase in UA concentration in the jugular vein blood compared to basal values (Fig 1). At the same time, the UA concentration of the portal vein blood remained the same as that measured 1 hour prior and was significantly lower than that observed in the jugular vein blood (2.43 ± 0.28 *vs*. 3.34 ± 0.52 mg/dL, respectively, *p* = 0.024). The results obtained suggest that after reaching a certain plasma urate threshold, the elimination of UA via the intestine starts occurring in pigs.

### Plasma UA concentrations in CKD pigs with induced HUA following oral uricase treatment

The 9/10 nephrectomy surgery was performed in pigs to create a model closest to that of a patient with the late stages of CKD. The surgical procedure and postoperative management did not seem to disturb the overall health status and growth of the pigs that underwent surgery, except during the first 6 days after surgery, where no weight gain was observed. Due to the presence of uricase, the nephrectomy did not increase plasma UA concentrations in the pigs (Table 1). To test the effectiveness of oral uricase treatment, five out of the eight operated pigs were selected based on elevated plasma creatinine levels and decreased creatinine clearance, which confirmed the development of advanced stages of CKD (Table 1).

**Table 1:** Biochemical parameters in plasma and urine of healthy and nephrectomized pigs.

Parameter	Healthy pigs, n = 3	9/10 nephrectomized pigs, n = 5
Plasma uric acid concentration (mg/dL)	1.56 ± 0.13	1.67 ± 0.24
Urinary uric acid excretion (mg/24 h)	129 ± 37	290 ± 48*
Urate clearance (mL/min)	4.38 ± 0.60	9.60 ± 2.24*
Plasma creatinine concentration (mg/dL)	1.34 ± 0.09	2.79 ± 0.88*
Urinary creatinine clearance (mL/min)	32.3 ± 16.3	18.8 ± 8.2

Data are presented as means ± SD.

Asterix (*) describes a significant difference between healthy and nephrectomized pigs.

A stable porcine model of HUA was achieved by frequent (every 30 min during a 7.5 h period, with 16 infusions in total) infusions with a UA suspension (5 mg/kg b.w.) into the jugular vein. This approach resulted in an approximately 2.6-fold increase in plasma UA concentrations during the first 12 h after beginning the UA infusion in CKD pigs, compared to that observed prior to the UA infusions (each pig served as its’ own control).

**Fig 2:**
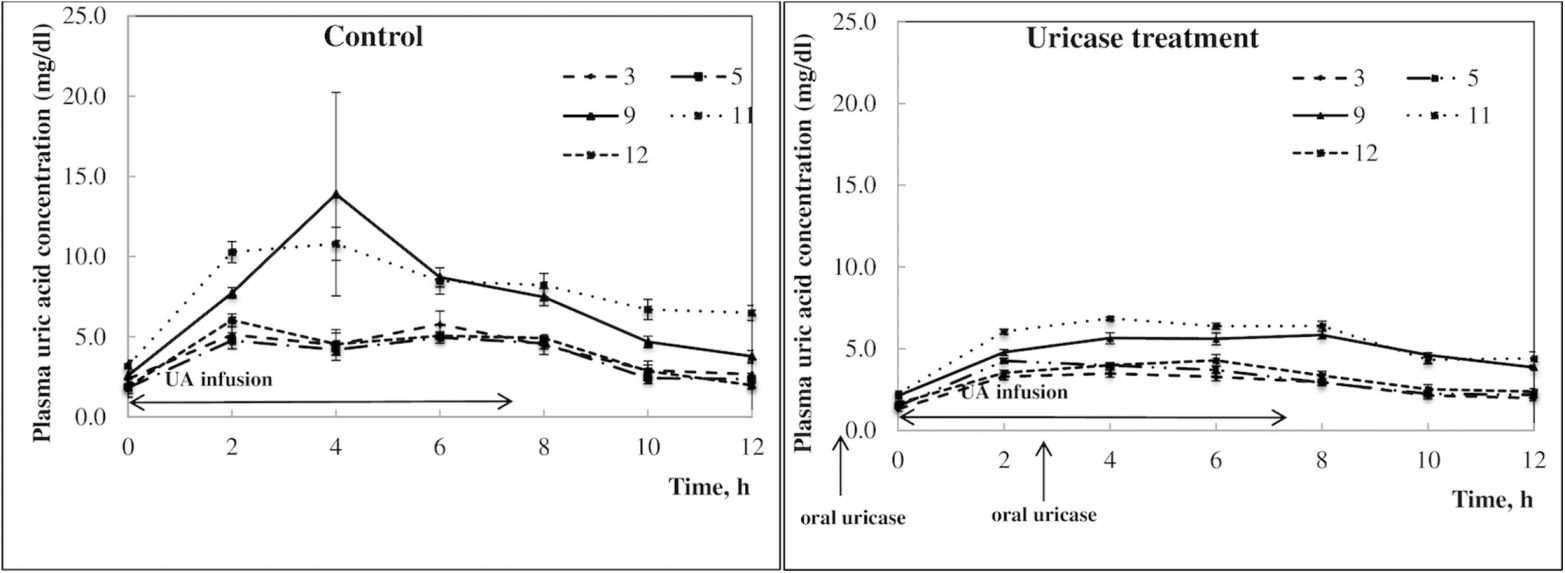
A, B. Plasma uric acid concentrations in 9/10 nephrectomized pigs i.v. infused with a uric acid suspension (every 30 min for 7.5 h, 5 mg/kg b.w.), without (control period–A) or with (uricase treatment period–B) oral uricase treatment during the first 12 h after beginning the uric acid infusions. Data are presented as means ± SD of both CT (control-treatment) and TC (treatment-control) sequences for each pig separately, *n* = 5 (each pig served as its’ own control).

The maximal plasma UA concentration in all pigs was observed 4 h after beginning the infusions (Fig 2A). The UA infusions were safe and well tolerated by the pigs and did not cause any GIT disturbances. The sequence of treatment/control periods had no significant effect on the resulting plasma UA concentrations.

Oral administration of uricase to pigs resulted in an approximately 1.3-fold decrease in plasma UA concentration in CKD pigs, 8 h after beginning the UA infusion, compared to the control period (Fig 2B). The most visible difference in plasma UA concentrations between control and enzyme-treatment periods was observed between 2–8 h after beginning the UA infusion. The AUC for the plasma UA concentration during the control period was 50.53 ± 2.80, 45.98 ±1.32, 91.31 ±9.08, 98.66 ±2.54 and 50.69 ±1.47 for pigs 3, 5, 9, 11 and 12, respectively. During the uricase treatment AUC for the plasma UA concentration decreased for each pig to values of 33.59 ± 0.69, 37.85 ± 0.63, 58.92 ± 0.85, 66.62 ± 0.81 and 39.36 ± 0.86 for pigs 3, 5, 9, 11 and 12, respectively. The pre-test that was performed to check the assumption of negligible carryover effects revealed no evidence of relevant carryover effects (T value -0.067, p = 0.948). A significant reduction in plasma UA concentration was observed during the treatment period in comparison to that observed during the control period, on the basis of the within-subject differences between control and treatment periods (T value 4.020, p = 0.03). The plasma UA concentrations during the control and enzyme-treatment periods reached baseline concentrations (~ 2 mg/dL) 18 h after beginning the UA infusions.

**Fig 3:**
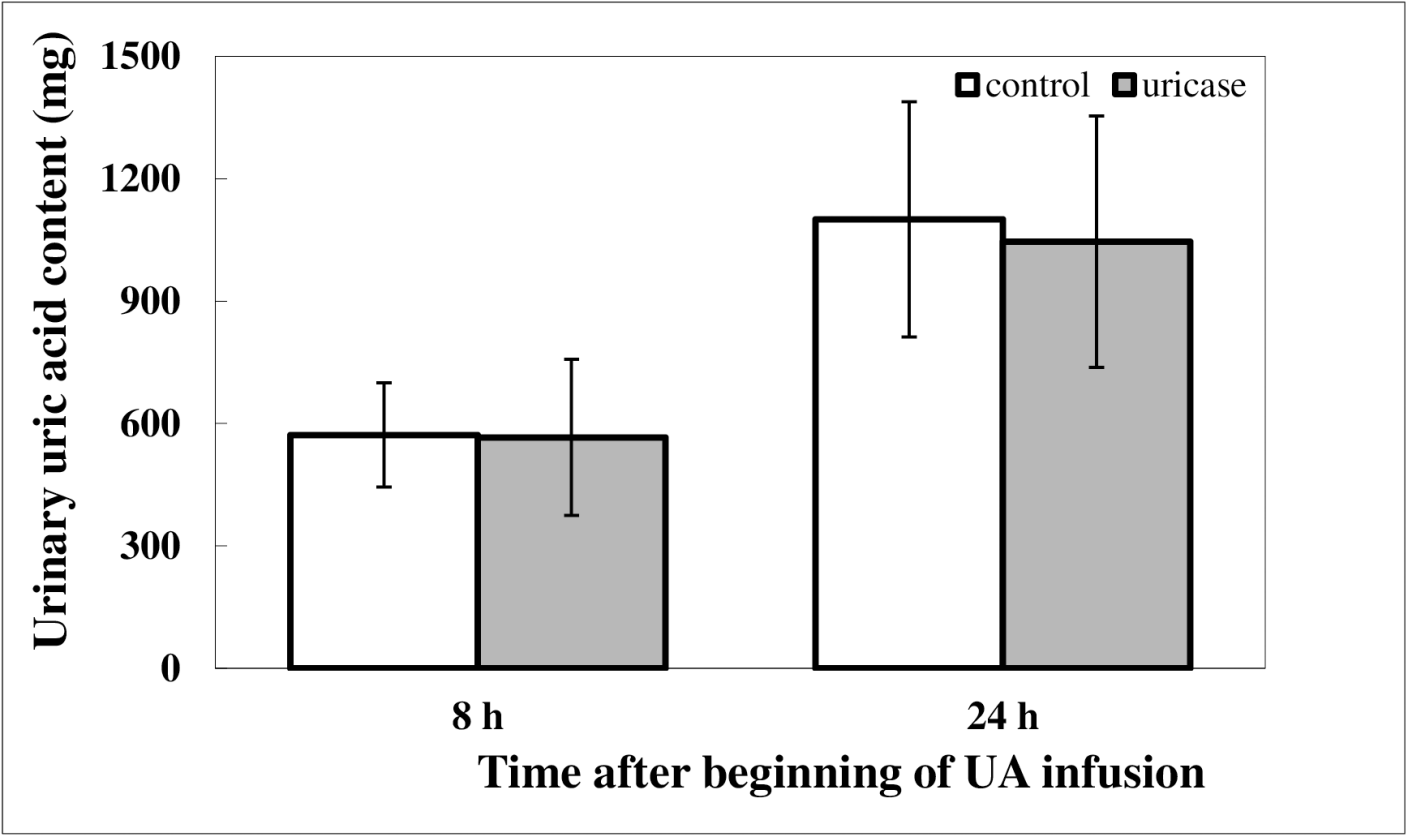
Urinary uric acid excretion in 9/10 nephrectomized pigs i.v. infused with a uric acid suspension (every 30 min for 7.5 h, 5 mg/kg b.w.), without (control period) or with (uricase treatment period) oral uricase treatment during the first 8 h and 24 h after beginning the uric acid infusions. Data are presented as means ± SD, *n* = 5 (each pig served as its’ own control).

Urinary UA concentrations are shown in Fig 3. Urinary UA excretion was similar in pigs during both sequences, in both study periods during the first 8 h and 24 h after beginning the UA infusions.

The creatinine and urate clearance were similar in pigs during both sequences, in both study periods and reached 23.8 ± 10.7 and 21.6 ± 18.8 mL/min during the control period, and 19.9 ± 7.0 and 21.3 ± 12.3 mL/min during the treatment period, respectively.

## Discussion

Hyperuricemia (HUA) is an abnormally elevated blood UA concentration in humans [5]. When the physiological saturation threshold for UA is exceeded, the formation and deposition of urate crystals (tophi) occurs in all tissues [11]. The crystals trigger acute arthritis, very often accompanied by excruciating pain and inflammation [12, 13]. Impaired renal elimination of UA causes more than 90% of all HUA cases [2]. HUA is strongly associated with a number of disorders, including kidney stones, obesity, diabetes, metabolic syndrome, hypertension, cardiovascular disease, cancer and gout [1, 6, 14]. Gout is one of the most common metabolic diseases in humans, the prevalence of which has nearly doubled over the last few years, affecting between 1–2% of the population in Western countries [15]. Moreover, the development of at least partial renal failure and chronic kidney disease (CKD) often occurs in conjunction with gout. Since UA is predominantly excreted by the kidneys, a drop in GFR increases the prevalence of HUA. Approximately half of the patients with CKD stages I to III become hyperuricemic and HUA is even more common in patients with CKD stages IV and V [2].

Most of the drugs targeted at lowering blood UA concentrations are focused on limiting hepatic UA synthesis or enhancing the renal excretion of UA. However, impaired renal function in CKD patients, where HUA results from the under-secretion, rather than overproduction of UA, increases the toxicity of drugs used to treat HUA. Consequently, patients with HUA and CKD are very often left untreated. Therefore, there is a need for the development of alternative therapies that would be effective in lowering serum UA concentrations in patients with CKD and on dialysis for the long-term management of HUA, without gout.

To date, a model of HUA has been developed only in rodents. Several reports have demonstrated that in rats HUA can be induced by a high-fat diet and starvation [16], dietary supplementation with purines [17] or administration of the uricase inhibitor—oxonic acid [18, 19]. Wu et al. [20] generated a mouse model of HUA with a targeted mutation at the urate oxidase locus that resulted in a 10-fold increase in blood UA concentration. These models led to both increased generation and decreased excretion of UA, urate crystal deposition and consequently, renal failure. They have been widely used for testing various therapies of HUA and gout, however they do not accurately reproduce the human pathology. For example, the harmful effects of urate crystals are more severe and occur much earlier in mice compared to humans [20]. Porcine models are much more reliable and more acceptable as models for humans in clinical research, due to the anatomical and physiological similarities between pigs and humans, especially with regards to the urinary system and digestive tract. In addition to similar multi-pyramidal kidneys (unlike rodents), humans and pigs also have comparable maximal urine concentration, GFR and total renal blood flow characteristics [21]. Unfortunately, one of the main disadvantages associated with the porcine model is the action of their own uricase enzyme (not present in humans), and thus, plasma UA concentrations in pigs are below 2 mg/dL. For this reason, development of a porcine model of hyperuricemia is challenging. Moreover, the excretion mechanism of urate also varies among species. For example, urate is actively reabsorbed in the tubular lumen in humans, resulting in very low fractional excretion of urate [22]. On the other hand, pigs and rabbits excrete more urate than is filtered through the glomerulus and thus have a fractional urate excretion in excess of the filtered load of urate [22].

As previously mentioned, our research aim was to develop a reliable pig model for investigation of the extra-renal elimination of UA through the intestine and to investigate the ability of orally administered uricase, from *Candida utilis*, in decreasing plasma UA concentrations in CKD pigs with induced HUA. Even the restriction of kidney function by 90% following 9/10 nephrectomy failed to increase plasma UA concentrations in the pigs used in the current study. However, the nephrectomized pigs demonstrated elevated plasma creatinine concentrations (Table 1), indicating reduced kidney function. In rats with renal failure following 5/6 nephrectomy, despite decreased urate clearance, plasma UA concentration also remained unchanged [23, 24]. Induced nephrectomy is a commonly accepted model of CKD, which mimics progressive renal failure after loss of renal mass in humans [25].

Unfortunately, we were not able to accurately predict the outcome of the nephrectomy surgery and thus we had to develop a crossover study design, with a minimum of four animals necessary for Study 2. Thus, the five pigs that successfully developed CKDh were sufficient for further inducement of HUA. In the current experiment, we increased plasma UA concentrations in the pigs by frequent (every 30 min for 8 h) i.v. infusions of a UA suspension into the peripheral blood, at a dose of 5 mg/kg b.w. We obtained a relatively stable porcine model of mild HUA (around 4 mg/dL) for 8 h (Fig 2). A similar 2-fold increase in plasma UA concentration was obtained in a rat model of mild HUA induced by dietary supplementation with oxonic acid [19].

In order to examine whether UA is eliminated via the intestine in pigs, as it is in humans, we compared the plasma UA concentration in the jugular vein blood, representing peripheral circulating UA concentrations with that in the portal vein blood, representing UA concentrations from the intestinal circulation, following the UA infusions. The results obtained clearly demonstrate that UA, after reaching a certain plasma urate threshold is also eliminated via the intestine in pigs, as it is in humans (Fig 1).

Uric acid handling is a very complex and dynamic process mediated by multiple specific transporters. In humans, blood urate is freely filtered in the kidney glomerulus [26]. Filtered urate is then reabsorbed in the proximal tubule by transporters URAT1 and GLUT9. Other transporters, such as OAT1-3, BCRP, NPT1, NPT4 and MRP4, are involved in the renal secretory transport of uric acid [27]. As a consequence, only about 3–10% of the filtered urate is eventually excreted in the urine [26].

Unfortunately, there is not much data on the molecular mechanisms of extra-renal excretion of uric acid. It has, however, been shown that intestinal secretion is the major extra-renal elimination pathway of UA [27]. Hosomi et al. [27] also showed that UA was excreted directly from the blood into the intestinal lumen, but the secretion varied in different parts of intestine (ileum > colon > jejunum). Nonetheless, the UA recovery was not complete, suggesting the existence of other routes of excretion, e.g. in the expired air or from the gut lumen [28]. One of the most important intestinal transporters seems to be BCRP, the activation of which resulted in decreased serum UA concentrations in hyperuricemic rats and the expression of which is much higher in the intestinal epithelial cells and hepatocytes than in the proximal tubular cells [27]. Genetic polymorphism in the BCRP gene has also been correlated with plasma UA concentrations and gout in humans [29]. The presence of human transporter homologues has also been confirmed in pigs [30]. Importantly, in patients with CKD there might be a compensatory mechanism promoting intestine uricolysis, which further increases the amount of UA released into the gut lumen [23]. It looks like the main reason for this is however the decrease in glomerular filtrate of uric acid, and not the tubular handling of uric acid [24]. The authors of the study which made use of nephrectomized rats with moderate CKD, noted a significant decrease in urinary excretion of uric acid, however plasma uric acid levels remain unchanged. The significant decrease in urinary uric acid excretion could be as a result of the increased expression of the BCRP transporter in the ileum. Together these results suggest that the enhancement of this elimination pathway may be effective as an alternative therapy for patients with renal failure.

Based on the above mentioned facts, we were interested in investigating the potential of oral uricase therapy for the enhancement of intestinal elimination of UA in patients with CKD and HUA. Uricase from *Candida utilis* was selected as the lead candidate for potential oral enzyme therapy due to its wide pH profile (6–8), which matches that of the small and large intestines, and based on results from previous *in vitro* studies [31]. Moreover, in our previous study we demonstrated that it is possible to reduce HUA with oral uricase therapy in a genetic mouse model of severe HUA and kidney failure (unpublished data). The results obtained in the present study proved this hypothesis in the pig model and confirmed that high concentrations of UA in the plasma can be reduced by oral administration of uricase from *Candida utilis*. The significant reduction in AUC of plasma UA concentration during the uricase treatment period compared to that observed during the control period (Fig 2), together with the restoration of plasma UA levels to baseline levels after 10–12 h and the same urinary UA excretion during the uricase treatment period and the control period (Fig 3) allow us to suggest that the decline in plasma UA concentration occurred due to enhanced gut elimination and not kidney excretion of UA. The identical kidney excretion of UA, independent of plasma UA concentration could suggest a limited GFR in the CKD pigs.

For several decades exogenous uricase has been i.v. administered to test its ability to decrease plasma UA concentrations, but there is a lack of data showing the impact of oral uricase therapy on plasma UA concentrations. Humans and higher primates do not possess uricase and are unable to metabolize UA, and thus, they are at high risk of developing HUA. Clinically approved uricase preparations include uricase from *Aspergillus flavus* known as rasburicase or recombinant uricase conjugated to polyethylene glycol (PEG)—pegloticase. Although, these preparations significantly reduce plasma UA concentrations and act faster than allopurinol, their use in the case of chronic HUA is very limited due to high antigenicity, rapid elimination from the plasma and induction of anti-PEG antibody production [32]. There have been attempts to create a uricase, encapsulated in an enzymosome, which increases its catalytic activity [3]. In contrast, oral administration of uricase should be neither immunogenic nor liver- or nephrotoxic, because it is not absorbed into circulation. It was assumed that uricase given orally would enhance intestinal secretion based on the UA gradient between the circulation and gut lumen. It has also been reported that HUA is treatable with the use of sevelamer, a non-absorbable hydrogel that adsorbs UA nonselectively in the GIT [33]. Nevertheless, more detailed studies are needed to estimate the kinetics of orally administered uricase.

Undoubtedly, the present study has certain limitations. The main limitation is the fact that the porcine model doesn’t fully mimic human physiology due to the presence of liver uricase in pigs. A further set of experiments should be performed to clarify the possible mechanisms underlying the results obtained in the present study. Both analysis of UA concentration in the portal vein of nephrectomized pigs and UA concentration in the stool should be performed to fully support the hypothesis that UA is eliminated via the intestine in CKD pigs with HUA. Moreover, while the present study is characterized by a short duration, in order to determine clinical relevance a long-term study needs to be conducted.

In summary, the results obtained from the present study in the porcine model suggest the possibility of decreasing plasma UA concentrations in patients with HUA and impaired renal function following oral therapy with microbial uricase, which possibly enhances UA elimination via the intestine. This approach may represent an alternative and safe therapy for the treatment of HUA in patients with attenuated GFR as strongly manifested in CKD.

## Supporting information

S1 Study Protocol Oral uricase eliminates blood uric acid in the hyperuricemic pig model. **Study protocol.** Original study protocol.
(DOCX)
