# Supplementary material for: Oral uricase eliminates blood uric acid in the hyperuricemic pig model
Source: PLoS One. 2017 Jun 8;12(6):e0179195. doi: 10.1371/journal.pone.0179195 (PMC5464639; doi:10.1371/journal.pone.0179195)
Supplement: S1 Study Protocol — Study protocol. Original study protocol. (DOCX) [file pone.0179195.s001.docx]

**Oral Uricase Eliminates Blood Uric Acid in the Hyperuricemic Pig Model**

**Study protocol**

**Aim of the study**

Uric acid (UA) is a poorly soluble final product of purine metabolism in humans. Most mammals express urate oxidase (uricase) which converts UA to the more soluble allantoin [1]. However, due to nonsense mutations in the uricase gene in humans, great apes and several other species, ‘uricase knockouts’, are unable to degrade UA. Under physiological conditions the majority of UA circulates in the plasma in a free form, as ionized urate salt. About two-thirds of the daily UA pool is primarily excreted by the kidneys, but more than 90% of this amount is reabsorbed by transporters in the proximal renal tubule [2]. Only 10% of the daily UA pool is then excreted from the kidneys via the urine. The remaining one-third is cleared through the gastrointestinal tract (GIT), where urate can be eliminated by gut bacteria in a process called uricolysis [3]. Plasma UA concentration depends on the balance between UA generation and excretion, as well as purine *de novo* synthesis, catabolism and turnover [4]. Both increased production of UA and/or its impaired excretion may lead to hyperuricemia (HUA), a condition defined by a plasma UA concentration above 6.8 mg/dL [5]. Due to the lack of uricase and the reabsorption of UA in the kidney proximal tubule, UA concentrations in humans are much higher in comparison to that of other mammals and moreover, they can be easily modified by diet. The consumption of alcohol, dietary purines (meat, seafood, offal) and products with high fructose content are possible risk factors for the development of HUA and gout. There is evidence to suggest that some genetic factors may also contribute to the development of HUA, specifically those involving polymorphisms in urate transporters [6].

Three main pharmacological treatment strategies are currently used in the treatment of HUA. The first treatment strategy is aimed at lowering UA generation and includes the use of xanthine oxidase inhibitors such as allopurinol and febuxostat. However, their effectiveness in lowering serum urate concentrations is limited, since only between 30-60% of patients receiving these therapies reach a plasma UA concentration of < 6 mg/dL [7]. Moreover, allopurinol is eliminated by the kidneys, and thus patients with chronic kidney disease (CKD) and thus impaired GFR may be at an increased risk of its toxicity [2]. The second treatment strategy is based on the use of uricosuric agents, such as probenecid and benzbromarone, which promote the excretion of UA in the urine. Unfortunately, these drugs can increase the crystallization of UA, inducing the formation of kidney stones and/or the development of hepatotoxic effects. The final treatment strategy includes the use of exogenous uricases, which metabolize UA to allantoin. Allantoin is 5 - 10 times more soluble than UA, and thus is more readily eliminated [4]. Clinical trials have shown that microbial uricase is more effective for the treatment of HUA and gout than allopurinol [8]. A recombinant form of uricase, rasburicase, is however characterized by a short half-life and high immunogenicity. Other uricase preparations, such as pegloticase, may be easily deactivated and physically unstable [3]. Thus, due to the undesirable biological properties and possible side effects of microbial uricase intravenous infusions, their use is very limited.

Thus, the majority of the currently approved therapies for HUA and gout are not recommended for patients with late-stage chronic renal disease. There is therefore a need for alternative, safe and well tolerated therapies which are effective in reducing plasma UA concentrations in patients with late stages of CKD.

**Research objectives:**

1. To develop a reliable pig model for investigation of the extra-renal elimination of UA through the intestine.
2. To investigate the ability of orally administered uricase, from *Candida utilis*, to decrease plasma UA concentrations in CKD pigs with induced HUA.

**Materials and methods:**

**Animals**

| **Species/Strain** | Pigs: ((Swedish Landrace X Yorkshire) x Hampshire) |
| --- | --- |
| **Sex** | Castrated males |
| **Age** | 10 ± 2 weeks at start of the experiment |
| **Weight** | 12 ± 3 kg |
| **Number** | 11 |
| **Id-N°** | Breeder identification numbers – to be included in study documentation |
| **Source** | Vindfälle 810, 268 68 Röstånga, Sweden |

**Sample size calculations:**

*For the initial investigation of the intestinal elimination of UA in healthy pigs* the sample size should be estimated for a two-sided t-test at α = 0.05 with 80% power, assuming delta = 75 and SD = 32. The SD of 32 was obtained from our previous study in which we also made use of a UA infusion to healthy pigs. The sample size for a two-sided t-test calculation according to Kraemer, H. C. and Thiemann, S. [9]:

$$n=\frac{{2\left( Z_{1-\alpha/2}+Z_{1-\beta} \right)}^{2}\sigma^{2}}{\delta^{2}}$$

The calculation yields a result of n=3, and this is the number of pigs that will undergo surgery to have a catheter inserted into the jugular vein and one into the portal vein.

*To create the pig model of CKD via nephrectomy surgery* the sample size should be estimated for a two-sided t-test at α = 0.05 with 80% power, assuming delta = 45 and SD = 32 using the formula given above. The calculation yields a result of n=8, and this is the number of pigs that will undergo 9/10 nephrectomy surgery.

*To investigate the ability of orally administered uricase, from Candida utilis, to decrease plasma UA concentrations in CKD pigs with induced HUA* the sample size should be estimated for a two-sided t-test at α = 0.05 with 80% power, assuming delta = 45, SD = 32 and a cross-over study design (one subject contributes to both arms) according to Kraemer, H. C. and Thiemann, S. [9]:

$$n=\frac{\left( Z_{1-\alpha/2}+Z_{1-\beta} \right)^{2}\sigma^{2}}{\delta^{2}}$$

The calculation yields a result of n=4, and this is the minimum number of CKD pigs that will be used in the cross-over investigation of the effects of uricase on plasma UA concentrations.

**Surgery:**

In all pigs the left external jugular vein will be catheterized using silicon tubing (Helix Medical Carpinteria, CA, USA) with an outer diameter of 1.64 mm and an inner diameter of 0.75 mm. The catheter will be exteriorized percutaneously on the dorsal side of the neck.

For investigation of the extra-renal elimination of UA through the intestine, the three healthy pigs will be catheterized with an additional catheter inserted into the portal vein, just prior to the entrance to the liver. The portal vein will be catheterized using silicon tubing (Silastic Laboratory Tubing, Dow Corning, Auburn MI, USA) with an outer diameter of 2.29 mm and an inner diameter of 1.27 mm.

In order to induce CKD in the pigs, 8 pigs will undergo 9/10 nephrectomy surgery. The nephrectomy will be achieved by renal tissue infarction. The main left renal artery will be completely ligated using sterile silk sutures which may lead to the blockage of blood flow to the entire left kidney. The right renal arterial branches will be ligated successively in order to achieve an obstruction to blood flow to 90 % of the right kidney.

Prior to surgery pigs will be fasted overnight and pre-medicated with azaperone (Stresnil, Janssen Pharmaceutica, Beerse, Belgium, 4.0 mg/kg i.m.). The pigs will be anaesthetized with 2-bromo-2-1.1.1-triflouroethane (Fluothan, Astra Läkemedel, Södertälje, Sweden), mixed with air and O_2_ as a carrier gas, at approximately 0.5 - 1 L/min in a close-circuit respiratory system (Komesaroff Medical Developments, Melbourne, Australia).

**Post-operative care:**

After the surgical procedures, for 3 days following surgery, the pigs will receive 1.5 mL of Buprenorphine (“Vetergesic”, Alstoe Ltd., York, UK). The pigs will be allowed a 2-week recovery period before the experiment commences.

**Feeding:**

Pigs will be fed twice daily (2 % of their b.w.) with cereal-based feed (“Morawski”, Żurawia, Poland), with a low calcium (0.09 %) and high fructose (20 %) concentration. The feed will be enriched with inosine (4 % of total food amount/day) to induce HUA. During enzyme treatment, the feed will also be supplemented with sodium bicarbonate (1 % of total food amount/day). Water supply will be limited and correspond to an amount of between 10-12 % of the pigs’ body weight. During the experimental period food and water intake will be measured daily. Pig body weight will be measured weekly.

**Experimental design:**

**Study 1:**

**1.** The initial study on the intestinal elimination of UA in healthy pigs (n=3) on two separate occasions with a 1-week interval between the two assessments.

**Study conditions:**

To reach saturation threshold for UA, the pigs will be intravenously infused, via the jugular vein catheter, with a UA suspension (40 mg/mL in 40 % glucose, pH 7.0, at dose of 5 mg/kg b.w.) every 30 min during a two hour period; thus the pigs will receive four infusions in total.

**Study 2:**

1. Estimation of the effects of the 9/10 nephrectomy surgery. The pigs that successfully developed CKD will be selected from the 8 pigs that underwent surgery (based on elevated plasma creatinine concentrations).
2. The two-period, two-sequence crossover study to investigate the ability of orally administered uricase, from *Candida utilis*, to decrease plasma UA concentrations in CKD pigs with induced HUA (minimal n=4).

**Study conditions:**

**Study periods: Control (C), Treatment (T)**

**Control:** Pigs will be i.v. infused with a UA suspension (40 mg/mL in 40 % glucose, pH 7.0 at dose 5 mg/kg b.w.) every 30 min for an eight hour period – with 16 infusions in total, in order to induce the HUA condition.

**Treatment:** Pigs will be i.v. infused with a UA suspension (40 mg/mL in 40 % glucose, pH 7.0 at dose 5 mg/kg b.w.) every 30 min for an eight hour period – with 16 infusions in total, in order to induce the HUA condition. Uricase from *Candida utilis* (25 % pure in sodium borate, specific activity ~25 units/mg) will be administered orally to each pig, together with the feed (18,000 Unit per portion), 1 h prior to and 3 h after beginning the UA infusions. To ensure enzyme stability in the stomach, the pigs will receive both food and water supplemented with sodium bicarbonate (1%).

**Study sequences: CT (Control Treatment), TC (Treatment Control).**

**Within-sequence interval: 1 day.**

**Wash-out period: 1 day.**

All CKD pigs will be assigned to the CT and then to the TC sequence and thus serve as both the treatment and control groups, such that each pig will serve as its own control.

**Sampling:**

**Study 1:**

Blood samples from both the jugular and portal veins will be collected into lithium-heparin tubes (BD Vacutainer®, Franklin Lakes NJ, USA, REF 367884) at baseline (before UA infusions) and thereafter repeatedly at 1 and 2 h during the UA infusion. Urine will be collected every 12 h during the 24 h period.

**Study 2:**

Blood samples from the jugular vein will be collected into lithium-heparin tubes (BD Vacutainer®, Franklin Lakes NJ, USA, REF 367884) at baseline (before UA infusions), and then at 2, 4, 6, 8, 10, 12, 16 and 24 h following the first UA infusion. Urine will be collected every 8 h during the 24 h period.

**Sample processing and analysis:**

**Blood samples:**

Blood samples collected into lithium-heparin tubes (BD Vacutainer®, Franklin Lakes NJ, USA, REF 367884) will be stored on ice before being centrifuged at 3000×g, for 15 min at 4ºC. Plasma will be collected and the samples will be stored at -20 °C until further analysis. Plasma UA and creatinine concentrations will be analyzed spectrophotometrically using the Uric Acid Assay Kit and QuantiChrom™ Creatinine Assay Kit, respectively (BioAssay Systems, Hayward, CA, USA), according to the manufacturer’s protocols.

**Urine samples:**

To prevent precipitation of UA salts, 1-2 ml of 8 M NaOH will be added to the urine collection containers. After each collection, aliquots (about 10 mL) of urine samples will be transferred into tubes and stored at –20 °C until performing the creatinine assay. UA concentration will be measured in fresh urine samples. Before analysis, urine samples will be diluted 1/10 with distilled H_2_O for UA and 1/20 with 7 mM NaOH for creatinine. Urinary concentrations of UA and creatinine will be measured spectrophotometrically using the Uric Acid Assay Kit or QuantiChrom™ Creatinine Assay Kit, respectively (BioAssay Systems, Hayward, CA, USA), according to the manufacturer’s protocols.

**References:**

1. Johnson RJ, Lanaspa MA, Gaucher EA. Uric acid: a danger signal from the RNA world that may have a role in the epidemic of obesity, metabolic syndrome and cardiorenal disease: evolutionary considerations. Sem Nephrology. 2011;31(5): 394-399.
2. Sah OSP, Qing YX. Associations between hyperuricemia and chronic kidney disease: a review. Nephrourol Mon. 2015;7(3): e27233.
3. Tan QY, Zhang JQ, Wang N, Yang H, Li X, Xiong HR, et al. Improved biological properties and hypouricemic effects of uricase from Candida utilis loaded in novel alkaline enzymosomes. Int J Nanomedicine. 2012;7: 3929-3938.
4. Pui CH. Rasburicase: a potent uricolytic agent. Expert Opin Pharmacother. 2002;3(4): 433-442.
5. Grassi D, Ferri L, Desideri G, Di Giosia P, Cheli P, Del Pinto R, et al. Chronic hyperuricemia, uric acid deposit and cardiovascular risk. Curr Pharma Des. 2013;19(13): 2432-2438.
6. Trifilio S, Gordon L, Singhal S, Tallman M, Evens A, Rashid K, et al. Reduced-dose rasburicase recombinant xanthine oxidase in adult cancer patients with hyperuricemia. Bone Marrow Transplant. 2006;37: 997-1001.
7. Gustafsson D, Unwin R. The pathophysiology of hyperuricaemia and its possible relationship to cardiovascular disease, morbidity and mortality. BMC Nephrol. 2013;14: 164.
8. Whelton A. Current and future therapeutic options for the management of gout. Am J Ther. 2010;17(4): 402-417.
9. Kraemer HC, Thiemann S. (1987) How Many Subjects? Statistical Power Analysis in Research. 1987.
